# 

**Published:** 1924-12

**Authors:** 


THE
### HOSPITAL and HEALTH REVIEW

*Established 1886* *No.* 1881. *Vol. LXXIII*

*New Series.* Vol. III. No. 39. *December,* 1924. Price Sixpence

CONTENTS

| | PAGE |
|---|---|
| The Final Vindication | 359 |
| The Smoke We Breathe | 360 |
| Notes and Comments :— "The Hospital and Health Review"—A Happy Return—The Public Medical Services—Reducing Temptation—Health in the School—Three Million Lepers—Reform of the Poor Law—A Museum of Tropical Medicine—"Rats!"—Dangers of Dust—Food Value of the Banana—Middle Class Health Insurance—Hospitals and Cooks—Graphic Health Teaching—Passing the Wart—Surprise Visits to Mental Hospitals—The Letter and the Spirit | 361 |
| Hospital Men of Mark: Brigadier-General Brownlow and Mr. Lancaster-Gaye | 365 |
| Electro-Therapeutics at Liverpool | 366 |
| Research at Great Ormond Street | 366 |
| Where London Takes the Air | 367 |
| Cripples at Biddulph Grange | 368 |
| Recovery of the London Hospitals | 369 |
| Quantity Statistics and Internal Control. By J. E. Stone, F.S.A.A., F.S.S. | 370 |
| Public Health Administration: Interview with Dr. Lyster | 371 |
| To Lighten a Burden | 372 |
| Progressive Rochdale | 372 |
| Hospital Progress and Finance | 373 |
| Zoning a Great City | 375 |
| A Miniature Town from Hospital Waste | 376 |
| Correspondence | 378 |
| Reviews of Books | 379 |
| Echoes of the Month | 384 |
| Hospital and Health News | 385 |
| Points from Hospital Reports | 386 |
| Recent Appointments | 386 |
| Hospital Needs and Hospital Appeals | 387 |

